# Comparative effectiveness of autologous particulate dentin graft for alveolar ridge preservation: a network meta-analysis of randomized controlled trials

**DOI:** 10.1186/s12903-025-07371-7

**Published:** 2025-12-03

**Authors:** Yixuan Zhang, Hong Yan, Jun Li, Shumin Ji, Can Cao

**Affiliations:** 1 Department of Stomatology, General Hospital of Northern Theater Command, Shenyang, 110016 Liaoning People’s Republic of China; 2 China Medical University, Shenyang, 110016 Liaoning People’s Republic of China; 3 Dalian Medical University, Dalian, 116044 Liaoning People’s Republic of China

**Keywords:** Autologous particulate dentin graft, Bone graft substitute, Alveolar ridge preservation, Alveolar ridge volume, New bone formation, Bone regeneration

## Abstract

**Objectives:**

This network meta-analysis (NMA) rigorously assessed the regenerative potential of autologous particulate dentin graft (ADG) in the context of alveolar ridge preservation (ARP), comparatively analyzing its performance against particulate xenogeneic (XG), allogeneic (ALG), alloplastic (APG) grafts, and blood clot healing (BCH).

**Materials and methods:**

A comprehensive review was undertaken utilizing recognized databases (PubMed, the Cochrane Central Library, Embase, Web of Science, CNKI, Wanfang, and VIP database) from 2015 to 2024, registered on PROSPERO (CRD42024572610). This NMA assessed radiographic parameters of alveolar ridges, measuring coronal width, buccal height, and lingual height, and evaluated new bone formation via histomorphometric analysis. Utilizing the random effects model to assess the efficacy of treatments, while the quality of the studies was evaluated using the ROB2 and GRADE criteria.

**Results:**

25 randomized controlled trials involving 710 patients and 830 tooth extraction sites were conducted. Both ADG and XG significantly preserved alveolar ridge volume compared to BCH. Specifically, ADG (MD = 1.67; 95% CI: 0.81, 2.54, *P* < 0.001) and XG (MD = 0.93; 95% CI: 0.20, 1.65, *P* = 0.012) demonstrated superior preservation of coronal alveolar ridge width change, with ADG showing an advantage over XG (MD = -0.75; 95% CI: -1.45, -0.05, *P* = 0.035). Similarly, both ADG (MD = 0.64; 95% CI: 0.21, 1.08, *P* = 0.004) and XG (MD = 0.68; 95% CI: 0.31, 1.04, *P* < 0.001) significantly maintained and buccal and lingual alveolar ridge height change compared to BCH.

**Conclusions:**

ADG effectively preserves alveolar ridge volume while demonstrating comparable new bone formation profiles with conventional bone graft substitutes.

**Supplementary Information:**

The online version contains supplementary material available at 10.1186/s12903-025-07371-7.

## Introduction

Alveolar ridge preservation (ARP) addresses the critical challenge of dimensional changes following tooth extraction. ARP offers a strategic approach to socket healing [1]. Unlike spontaneous blood-clot healing (BCH), ARP maintains bone tissue structure through precise surgical intervention [2], preserving critical hard and soft tissue volumes. This method reduces post-extraction bone resorption, establishing an optimal biological substrate that significantly improves the potential for successful dental implant placement [3]. ARP involves the strategic application of bone graft substitutes to maintain the anatomical structure and physiological function of the alveolar ridge [4, 5].

The choice of bone graft substitutes for ARP is pivotal in the process of regenerating bones. Among these materials, particulate grafts are particularly advantageous [6], as they are more suitable for use in irregular extraction sockets, where their shape and size can enhance stability and integration. Grafts could significantly influence both the volume and the bone remodeling process [7, 8], specifically the velocity at which novel bone develops following the resorption of the graft substance. This effectiveness could be ascribed to their unique biological properties, including osteogenesis, osteoinduction, and osteoconduction [9]. Autogenous bone grafts are commonly acknowledged as the gold standard in bone regeneration owing to their non-immunogenic properties and predictable osteogenesis [10]. However, the necessity for a secondary surgical site can result in increased morbidity and extended recovery times [11]. Additionally, the rapid absorption rate of autogenous grafts may limit their application as an ideal bone grafts for ARP. In contrast, allogeneic particulate grafts (ALG) exhibit potential osteoinductive properties and remove the necessity for an additional surgical location [12], yet they carry inherent risks of immunogenicity and disease transmission. Concerns regarding the origin of ALG remain prevalent among many patients. Xenogeneic particulate grafts (XG) are readily available; however, they may provoke inflammatory responses and are limited to osteoconductive properties as a temporary scaffold [13, 14]. Alloplastic particulate grafts (APG) present a non-immunogenic alternative that poses no risk of disease transmission, but they often encounter similar challenges related to absorption and predictability [15]. The absorption rates of both XG and APG may impede volume maintenance and bone remodeling processes, which ultimately affect the effectiveness and efficiency of ARP.

Confronting the multifaceted challenges of existing bone grafting strategies, autologous particulate dentine grafts (ADG) emerge as a transformative biomaterial solution [16], representing a paradigmatic shift in regenerative approaches. Combining low-cost availability with exceptional bone regeneration potential, ADG addresses these challenges effectively [17, 18]. Composed primarily of type I collagen fibers and hydroxyapatite, this groundbreaking graft not only promotes regeneration of bones but also acts as a delivery system for vital growth factors. The demineralized dentine matrix within ADG is particularly valuable due to its richness in collagen and growth factors like bone morphogenetic proteins, which enhance its bone induction capacity [19]. Additionally, the autologous nature of ADG contributes to greater patient acceptance, as it minimizes concerns about immune rejection [20, 21]. Furthermore, because ADG is not subjected to high-temperature processing or complex treatments, it retains a rich supply of growth factors, providing superior osteoinductive properties, making it an ideal material for ARP. Nonetheless, the utilization of ADG derived from the patient’s own dental structures prompts apprehensions regarding the probability of diminished absorption rates within the body, or even the potentiality of non-absorption altogether. This could lead to worries about the effective space for new bone generation, which may impact the overall success of the graft. Despite these concerns, ADG minimizes the risk of immune rejection, reduces complications, and simplifies the graft procurement process, ultimately alleviating patient stress and surgical trauma [22].

Recent clinical studies have assessed the performance of ADG in ARP [23, 24], systematically comparing it with XG, ALG, APG, and BCH methods. This comprehensive investigation strategically evaluates critical dimensional and biological indicators, including coronal alveolar ridge width, buccal and lingual alveolar ridge height, and new bone formation. Coronal alveolar ridge width critically influences implant size selection and surgical planning, serves as a pivotal indicator for preoperative clinical assessment. Common buccal defects following tooth extraction result in differential changes in buccolingual bone height, which directly influence implant feasibility and long-term success rates [25–27]. The rate at which new bone is formed is intricately linked to the subsequent rates of bone integration and serves as a crucial metric for assessing the biocompatibility and osteogenic activity of grafts [28].

Hence, this network meta-analysis (NMA) aims to evaluate the regenerative potential of ADG compared to conventional grafts, focusing on changes of volume and new bone formation. By integrating direct and indirect evidence from XG, ALG, APG, and BCH, the study assesses ADG’s unique biomechanical and biological characteristics and its potential to address the limitations of current grafting strategies, providing insights into bone regeneration mechanisms in ARP.

## Materials and methods

### Protocol registration

This network meta-analysis (NMA) was conducted according to the PRISMA-NMA guidelines [29] and registered on PROSPERO (CRD42024572610).

### Search strategy and data collection

A comprehensive search was undertaken across PubMed, the Cochrane Central Library, Embase, Web of Science, CNKI, Wanfang, and VIP database for relevant studies. The list of parameters encompassed keywords and MeSH terms such as “xenograft”, “alveolar ridge preservation”, “autologous dental material”, and “autologous dentin graft”. Furthermore, a thorough manual search was conducted to identify additional pertinent studies by examining the bibliographies of the selected articles, as well as grey literature sources (Google Scholar and the DANS EASY Archive). The exhaustive search methodology is elaborated upon in Supplementary Table S1. The findings were confined to studies involving humans and RCTs. Two investigators, S.M.J and J.L, meticulously examined all titles and summaries from the electronic search to ascertain their compliance with the established criteria, subsequently evaluating the eligibility of the full-text articles independently. Disputes were addressed through discussion or by seeking the insights of an additional researcher. The process of data extraction was conducted utilizing an established format (Y.X.Z, H.Y). The agreement was assessed using Cohen’s kappa coefficient (κ ± ASE). The information extracted encompassed the features of the research, demographics of the participants, reason for extraction, smoking habit, specifics of the treatment, comparison groups, outcomes, and the duration of follow-up. The data and information that were not referenced or not possible to obtain have been denoted as “NA”, signifying “not available”. For data that have not been publicly reported, attempt to contact the authors to obtain the original data to ensure data completeness.

### Research question and eligibility criteria

The central inquiry was articulated as follows: “In healthy adults requiring alveolar ridge preservation after atraumatic tooth extraction, how does ADG compare with XG, ALG, APG and BCH in terms of alveolar ridge volume changes and new bone formation percentage?”.

We used PICOS to define inclusion criteria: Patients (P): Patients in good health getting atraumatic extractions of teeth, resulting in alveolar ridge defects, and subsequently receiving either alveolar ridge preservation treatment or allowed to heal only via blood clot formation. Intervention (I): Atraumatic extraction sockets were filled with autologous particulate dentin. Comparator (C): Atraumatically extracted sockets were filled with xenogeneic particulate graft, allogeneic graft, alloplast graft or only blood clot healing. Outcomes (O): Primary outcomes included radiological assessment (CBCT/X-ray) regarding alterations in the volume of the alveolar ridge, specifically in terms of curved horizontal and vertical height, while secondary outcomes focused on histomorphometric evaluation of new bone formation percentage. The measurements of the horizontal width and vertical height of the alveolar bone were obtained through CBCT or X-ray, utilizing images taken immediately before or after tooth extraction (baseline) and again at the 3–6-month recuperation interval. Study Design (S): Exclusively RCTs featuring a follow-up duration ranging from 3 to 6 months were considered for inclusion.

The criteria for exclusion were delineated as follows: Researches were not RCTs, such as pilot studies, case reports and systematic reviews; Preclinical in vitro or animal studies; Bone dimensions were measured with a periodontal probe during operations; Studies featuring insufficient data or data that could not be obtained.

### Quality assessment (GRADE) and risk of bias

The reliability of evidence was evaluated through CINeMA (Confidence in Network Meta-Analysis) methodology [30]. Based on the GRADE framework, five dimensions were considered, including “risk of bias”, “inconsistency”, “indirect evidence”, “imprecision”, and “publication bias”. The quality of evidence was later categorized as high, moderate, low, or very low, based on the assessment of these variables [31].

Two independent evaluators appraised the quality and potential for bias of the included papers utilizing the Cochrane Collaboration’s Risk of Bias assessment (ROB2). Studies were categorized as exhibiting “low,” “some concern,” or “high risk” of bias across many categories. Discrepancies in evaluations were rectified through discussion or contact with an additional evaluator.

### Data synthesis strategy

Stata 17 was used to perform statistical analysis, employing network meta-analysis packages st0410, st0411, and st0156-2. Continuous outcomes were reported as mean differences (MD) or standardized mean difference (SMD) with a corresponding 95% CI. Due to variations in the staining methods used to process bone samples across studies, the analysis of new bone formation data employed SMD to reduce inter-study heterogeneity. In contrast, for the remaining three outcome indicators, where the measurement methods were consistent across studies, MD was used exclusively. When only the 95% confidence interval (CI) was reported, the standard deviation (SD) was derived using the following formula: $\:SD=\sqrt{n}*\frac{{CI}_{97.5\%}-{CI}_{2.5\%}}{2*1.96}$. To address variability in measurement positions across studies, we consistently selected data points closest to the alveolar ridge crest for horizontal width analysis.

Within the framework of NMA, the node-splitting approach is employed to evaluate the consistence between direct and indirect evidence. Evidence was regarded as consistent whenever *P* > 0.05, and the conclusions derived from consistency algorithms were viewed as valid. The loop-specific methodology was employed to demonstrate discrepancies when *P* < 0.05. Heterogeneity among studies was analyzed using τ^2^ and I^2^. In order to assess the possibility of publication bias, we intended to employ comparison-adjusted funnel plots for visual analysis alongside Egger’s test.

## Results

### Study selection and Article characteristics

**Fig. 1:**
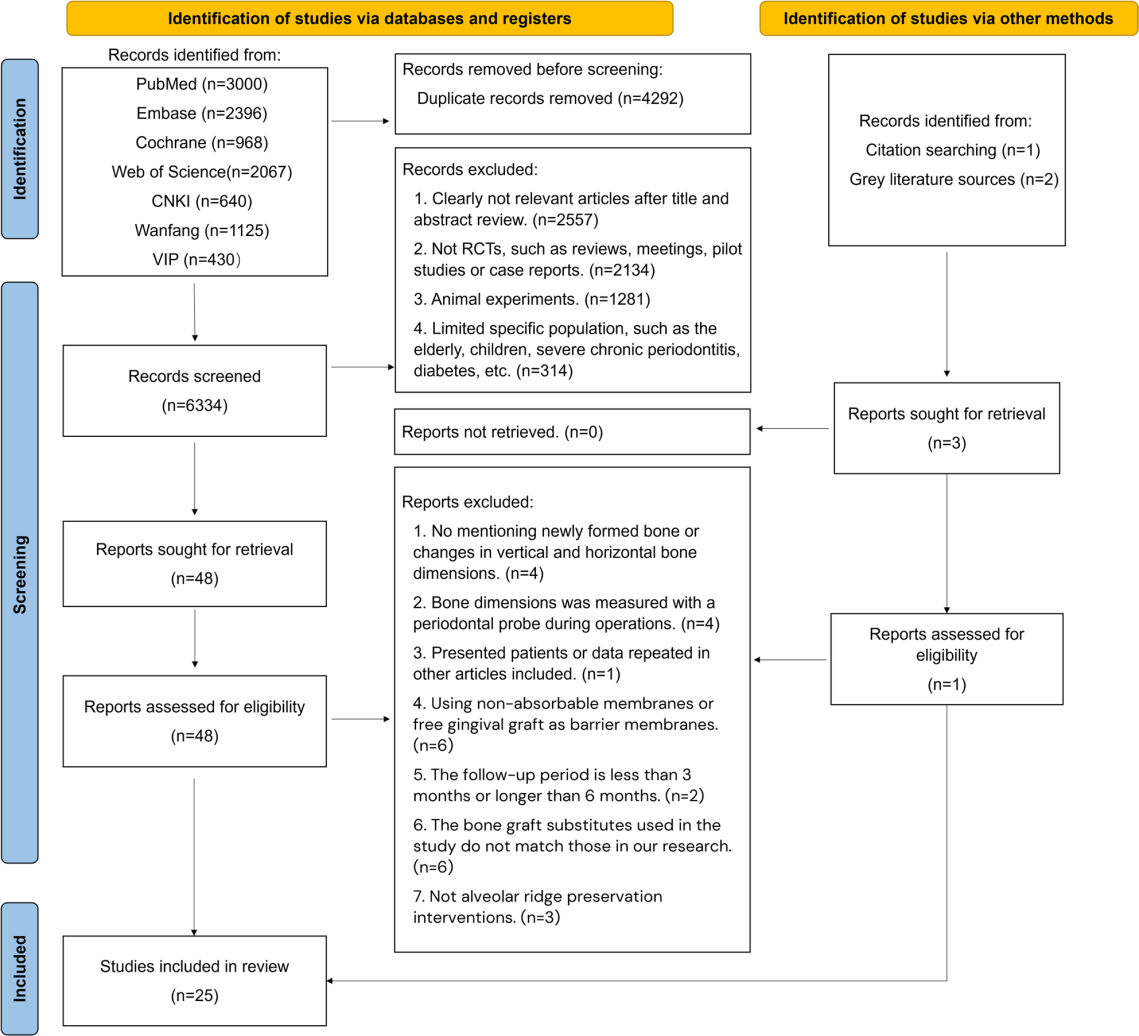
PRISMA flow diagram

Researchers have undertaken an exploration within PubMed, the Embase Database, the Cochrane Library, Web of Science, CNKI, Wanfang, and VIP database and grey literatures to identify prospective studies. The internet-based search yielded an aggregate of 10,626 publications (PubMed: 3000, Embase: 2396, Cochrane: 968, WOS:2067, CNKI: 640, Wanfang: 1125, VIP: 430). 4292 publications were eliminated due to duplication, and 6334 publications had their titles and abstracts closely examined. The agreement for the inclusion of studies was considered excellent (κ = 0.85 ± 0.07).After going through the full text, 25 studies [32–56] were found to be suitable for inclusion in this systematic review **(**Fig. 1**)**, involving 710 patients and 830 sites of tooth extraction were incorporated. Table 1 provides a comprehensive overview of the characteristics of the research projects that were incorporated, and Supplementary Table S2 shows the reasons of excluded studies. The outcomes data is available in Supplementary Table S3.

**Table 1:** Basic characteristics of the included studies

Study	Study design	Reason for extraction	Graft sites	Age(yr)	Smoking habit N/Y	Test Group				Control Group				Follow-up(mo)	Main outcomes
						No. patients*	No. sockets*	M/F	Graft materials	No. patients*	No. sockets*	M/F	Graft materials		
Abellán et al.,2022 [48]	RCT	an absence of healthy dental structure	maxillary or mandibular first or second molar	44.84	17/4	NA (10)	NA (10)	3/7	ALG	NA (11)	NA (11)	5/6	XG	5	①,②,③
Barone et al.,2016 [34]	RCT	decay/endodontic failure/fracture	premolar and molar	47.4	75/15	60(60)	60(60)	24/36	XG	30(30)	30(30)	12/18	BCH	3	③
Cadenas-Vacas et al.,2021 [44]	split-mouth RCT	caries, trauma, crown fracture, root fracture, etc.	uniradicular or biradicular teeth	51	12/0	12(12)	12(12)	5/7	APG	12(12)	12(12)	5/7	XG	3	③
Carlos et al., 2017 [37]	RCT	caries, fracture, endodontic failure	non-molar tooth	44.25	18/2	10(10)	10(10)	4/6	ALG	10(10)	10(10)	6/4	XG	6	③
Casarez-Quintana et al.,2021 [50]	RCT	NA	non-molar	55	NA	27(23)	27(23)	NA	XG	25(21)	25(21)	NA	ALG	3	③
Gabay et al.,2022 [51]	RCT	NA	premolar, canine or incisor	62.23	29/1	15(14)	15(14)	6/9	XG	15(14)	15(14)	5/10	BCH	6	③
G-U Jung et al., 2018 A [38]	RCT	root fracture, caries, endodontic failure, periodontitis	premolar and molar	47.25	NA	10(8)	10(8)	5/3	ADG	10(8)	10(8)	4/4	XG	4	①,②
Guarnieri et al.,2017 [35]	RCT	NA	premolar or molar	20–63	12/5	8(8)	8(8)	6/2	XG	9(9)	9(9)	5/4	BCH	4	③
Hussain et al.,2023 [54]	RCT	NA	maxillary nonmolar	35.6	29/0	16(14)	16(14)	NA	ADG	16(15)	16(15)	NA	BCH	4	①,②
Jung et al.,2018B [39]	split-mouth RCT	NA	molar or premolar	NA	NA	24(18)	24(18)	NA	XG	24(18)	24(18)	NA	BCH	6	①,②
Kim et al.,2024 [56]	RCT	Periodontal, fracture, endodontic	anterior, premolar and molar	64.2	NA	18(17)	18(17)	9/8	XG	18(17)	18(17)	14/3	BCH	6	①,②
Lim et al.,2019 [41]	RCT	periodontal/fracture/endodontic	molar	54.2	NA	22(21)	22(21)	16/5	XG	11(8)	11(8)	5/3	BCH	4	①,②,③
MacBeth et al.,2022 [52]	RCT	trauma, periodontitis, endodontic complication or unrestorable caries	maxillary single rooted incisor, canine or premolar tooth	32	28/0	14(14)	14(14)	NA	XG	14(14)	14(14)	NA	BCH	3	①,②
Machtei et al.,2019 [42]	RCT	NA	premolar, canine or incisor	63.9	24/9	11(11)	11(11)	NA	XG	11(10)	11(10)	NA	BCH	4	③
Candrli´et al,2022 [49]	RCT	periodontitis and crown or root fracture	anterior, premolar and molar	36.9	NA	21(20)	21(20)	7/13	APG	20(20)	20(20)	8/12	XG	6	③
Nunes et al.,2018 [40]	split-mouth RCT	NA	upper front teeth	39–65	15/0	15(15)	15(15)	4/11	APG	15(15)	15(15)	4/11	BCH	6	①,②
Ogui´c et al.,2023 [55]	RCT	hopeless tooth	maxillary incisors, canines, and premolars	54	NA	20(20)	20(20)	13/7	ADG	17(17)	17(17)	5/12	XG	4	①,③
Pang et al., 2017 [36]	RCT	hopeless tooth	anterior, premolar and molar	52	27/0	15(15)	21(21)	8/7	ADG	12(9)	15(12)	3/6	XG	6	③
Sadeghi et al., 2016 [32]	RCT	NA	Single rooted teeth	35.5	NA	10(10)	10(10)	NA	ALG	10(10)	10(10)	NA	XG	4–6	③
Saito et al.,2021 [45]	RCT	NA	maxillary or mandibular premolars or molars	53.5	NA	24(22)	24(22)	9/15	APG	21(17)	21(17)	7/14	ALG	4	①,②,③
Santos et al., 2021 [46]	RCT	mono- and multiradicular teeth	Anterior and posterior	59.1	48/4	26(26)	34(34)	11/15	ADG	26(26)	32(32)	10/16	XG	6	③
Scheyer et al.,2016 [33]	RCT	NA	first premolar-first molar	18–70	40/14	19(19)	19(19)	NA	XG	21(21)	21(21)	NA	ALG	6	③
Stumbras et al.,2020 [43]	RCT	Endodontic, fracture, periodontal, caries	anterior maxilla	52.5	19/1	11(10)	11(10)	2/8	XG	12(10)	12(10)	3/7	BCH	3	③
Yu¨ceer-C¸etiner et al., 2021 [47]	RCT	NA	NA	31–62	NA	NA	20(20)	NA	ADG	NA	16(16)	NA	BCH	3	③
Zampara et al.,2022 [53]	RCT	NA	anterior, premolar and molar	45–70	NA	8(8)	8(8)	NA	XG	8(8)	8(8)	NA	BCH	3	③
						8(8)	8(8)	NA	ALG	8(8)	8(8)	NA	APG	3	③

*RCT* Random controlled trail, *BCH* Blood clot healing, *XG* Xenogeneic graft, *ADG* Autologous dentin graft, *ALG* Allogeneic graft, *APG* Alloplastic graft, *NA* Not available

①: Coronal ridge width changes; ②: Buccal and lingual ridge height changes; ③: new bone formation

^*^The number before the parentheses represents the initially included sample size, while the number inside the parentheses represents the final sample size after excluding lost to follow-up

### Quality assessment and risk of bias

The confidence of evidence was assessed using the CINeMA system. The comparisons in this network meta-analysis showed moderate and low rates of confidence. The details of the confidence of evidence are provided in Supplementary Table S4. Inter-rater reliability for the GRADE was evaluated using Cohen’s kappa coefficient κ = 0.71 ± 0.20. The analysis of inter-study heterogeneity is presented in Supplementary Table S5.

**Fig. 2:**
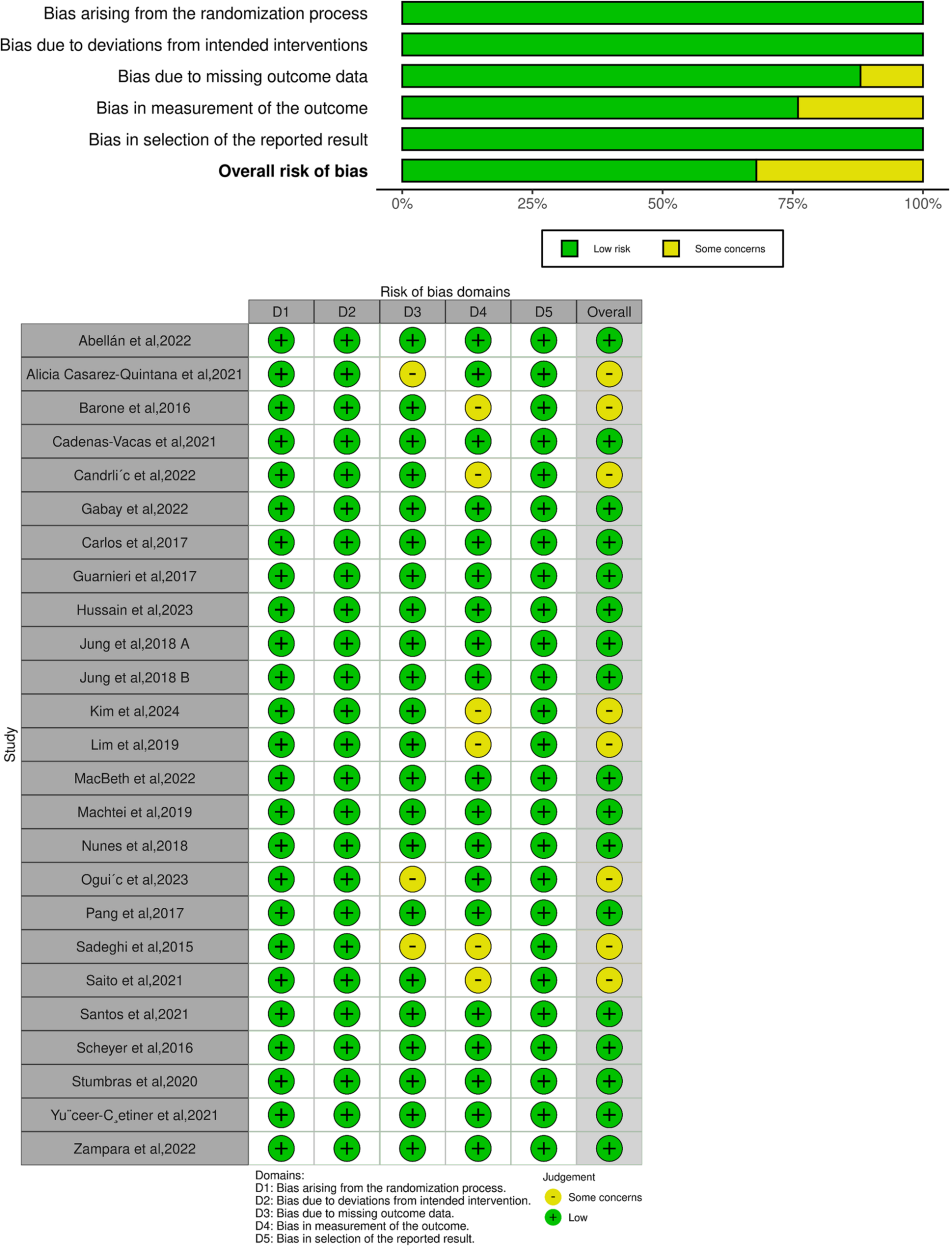
Summary of the included studies’ overall risk of bias. Green, yellow, and red indicate low, some concern, and high risk of bias, respectively

The risk of bias of the included studies was evaluated, and the results are shown in Fig. 2. Inter-rater reliability for the risk of bias assessment using the ROB2 tool was evaluated using Cohen’s kappa coefficient (κ = 0.74 ± 0.14) Regarding risk of bias assessment, 3 studies were rated as “some concerns” in the domain of “Missing outcome data”, as the loss to follow-up exceeded 5% of participants. Additionally, 6 studies were judged as “some concerns” in “measurement of the outcome”, since the outcome assessors were not blinded during the trials, which could potentially introduce detection bias. Due to the nature of surgical interventions, complete blinding of participants and personnel was not feasible, potentially introducing performance bias. This limitation was noted but not considered a critical flaw given the objective nature of the primary outcomes. Risk of bias showed mixed results with an overall low-medium risk of bias.

### Coronal alveolar ridge width changes

**Fig. 3:**
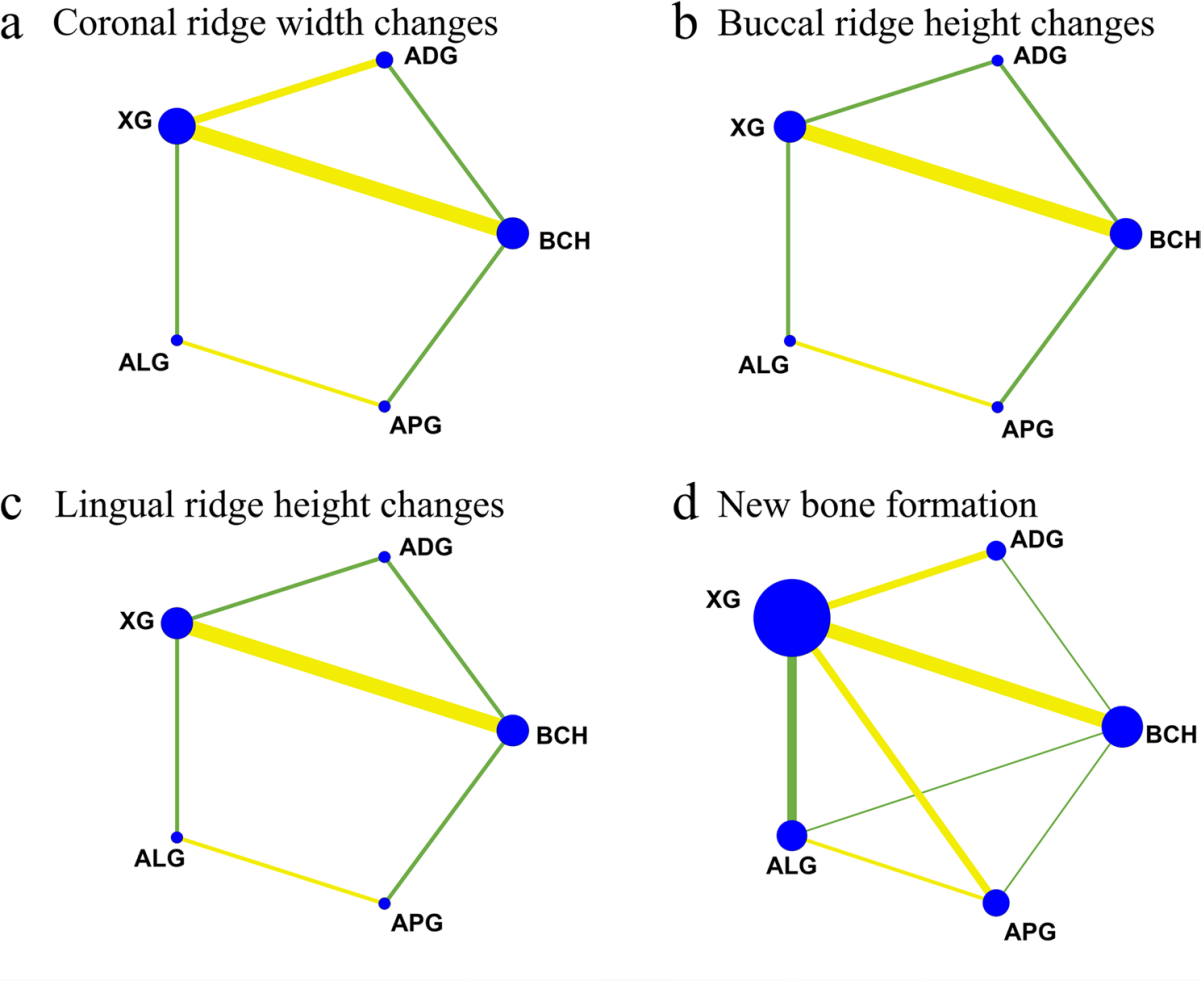
Network plot for coronal ridge width changes (**a**), buccal ridge height changes (**b**), lingual ridge height changes (**c**), and new bone formation (**d**). BCH: blood clot healing; XG: xenogeneic graft; ADG: autologous dentin graft; ALG: allogeneic graft; APG: alloplastic graft

**Fig. 4:**
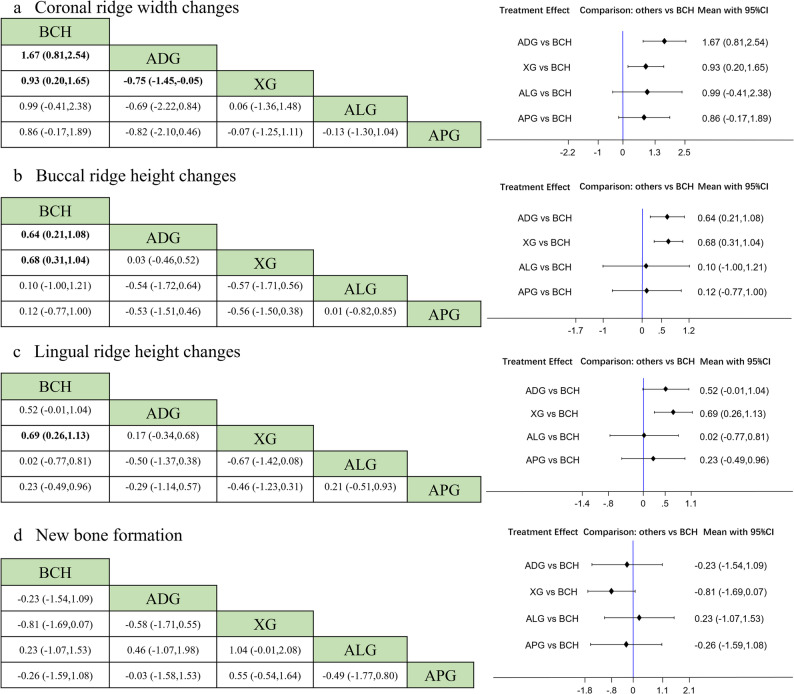
Forest plot for coronal ridge width changes (**a**), buccal ridge height changes (**b**), lingual ridge height changes (**c**), and new bone formation (**d**). The 95%CI contained the null value 0, suggesting that there were no statistically significant differences. BCH: blood clot healing; XG: xenogeneic graft; ADG: autologous dentin graft; ALG: allogeneic graft; APG: alloplastic graft

**Fig. 5:**
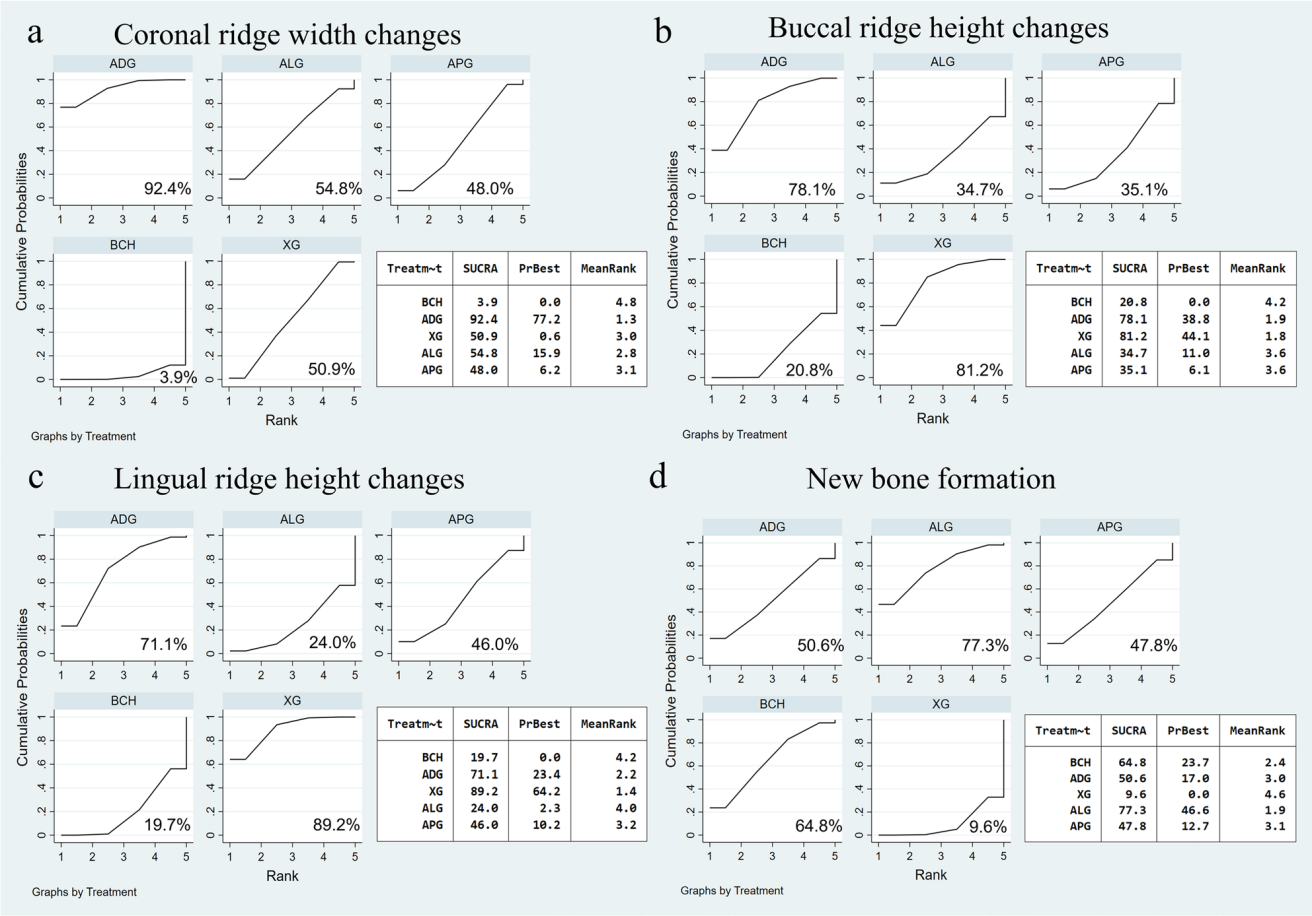
Plots of the surface under the cumulative ranking curve (SUCRA) for coronal ridge width changes (**a**), buccal ridge height changes (**b**), lingual ridge height changes (**c**), and new bone formation (**d**). The closer the SUCRA value is to 100%, the better the clinical effect will be. BCH: blood clot healing; XG: xenogeneic graft; ADG: autologous dentin graft; ALG: allogeneic graft; APG: alloplastic graft

The network diagram is presented in Fig. 3a. This analysis involved 300 sites from 10 RCTs. The size of each node represents the number of patients included in studies featuring that device. The thickness of the lines connecting the nodes is proportional to the number of head-to-head studies in each comparison. The densest comparison was BCH vs. XG, contributing 4 trials. And no head-to-head trials were available for ADG vs. ALG, ADG vs. APG, XG vs. APG, and BCH vs. ALG, so these estimates rely on indirect evidence. The evidence network consisting of ten studies formed a pentagonal closed loop structure, and no notable inconsistency was observed (Supplementary Table S6). Network meta-analysis results showed that compared to the BCH group, both ADG (MD = 1.67, 95% CI: 0.81, 2.54, *P* < 0.001) and XG (MD = 0.93, 95% CI: 0.20, 1.65, *P* = 0.012) significantly reduced horizontal alveolar ridge bone resorption (Fig. 4a), and ADG showed better result than XG (MD=−0.75, 95%CI: −1.45, −0.05, *P* = 0.035). There was no statistically significant difference between other groups because the confidence interval includes the null value. (*P* > 0.05). The cumulative ranking (Fig. 5a) indicated that all interventions were more efficacious than BCH in preserving alveolar ridge bone, with ADG showing the highest probability of being the best treatment, followed by ALG, XG, APG.

### Buccal alveolar ridge height changes

The network diagram for vertical buccal crest height changes is presented in Fig. 3b. This analysis involved 263 sites from 9 RCTs. The most extensively studied comparison was BCH vs. XG, supported by four trials. There were 6 direct comparisons. A pentagonal closed loop was observed among interventions, with no significant inconsistency in the results (Supplementary Table S6). Network meta-analysis results showed that compared to the BCH group, XG (MD = 0.68, 95% CI: 0.31, 1.04, *P* < 0.001) and ADG (MD = 0.64, 95% CI: 0.21, 1.08, *P* = 0.004) significantly reduced bone resorption on the buccal sides. However, there was no statistically significant difference between other groups since the confidence interval encompasses the null value (Fig. 4b). The cumulative ranking (Fig. 5b) indicated the effectiveness ranking of different materials for ARP as follows: XG, ADG, APG, ALG, while BCH demonstrated the lowest efficacy.

### Lingual alveolar ridge height changes

The treatment network comprised five interventions across 9 randomized trials, totaling 263 sites and reporting changes in lingual–palatal alveolar bone height. BCH vs. XG was the densest link (four trials), whereas the other head-to-head comparisons were each informed by a single study. The network diagram demonstrated that a closed loop was formed among the groups of comparisons (Fig. 3c), and there was no inconsistency can be seen (Supplementary Table S6). According to the forest plot, the comparison between XG and BCH was statistically significant (MD = 0.69, 95%CI: 0.26, 1.13), indicating XG could significantly reduce the bone resorption on the lingual-palatal side. The comparison between other groups had no statistical significance (Fig. 4c). The effectiveness ranking (Fig. 5c) of different materials for ARP was as follows: XG, ADG, APG, ALG, and BCH.

### New bone formation

Across 20 randomized trials, the network covered five interventions; one trial had a four-arm design [53]. The densest link was BCH vs. XG, with 7 direct studies, while ADG vs. ALG and ADG vs. APG lacked direct head-to-head data and were informed only by indirect evidence. Among them, one of the articles is a four-arm study [53]. The network diagram of treatment approach comparisons is presented in Fig. 3d. Analysis of the forest plot, all comparisons crossed the line of no effect, indicating no statistically significant differences between treatments (Fig. 4d). Based on the SUCRA score, ALG had the highest probability of being the optimal treatments, following BCH, ADG, APG, and XG had the lowest score (Fig. 5d).

### Publications bias

**Fig. 6:**
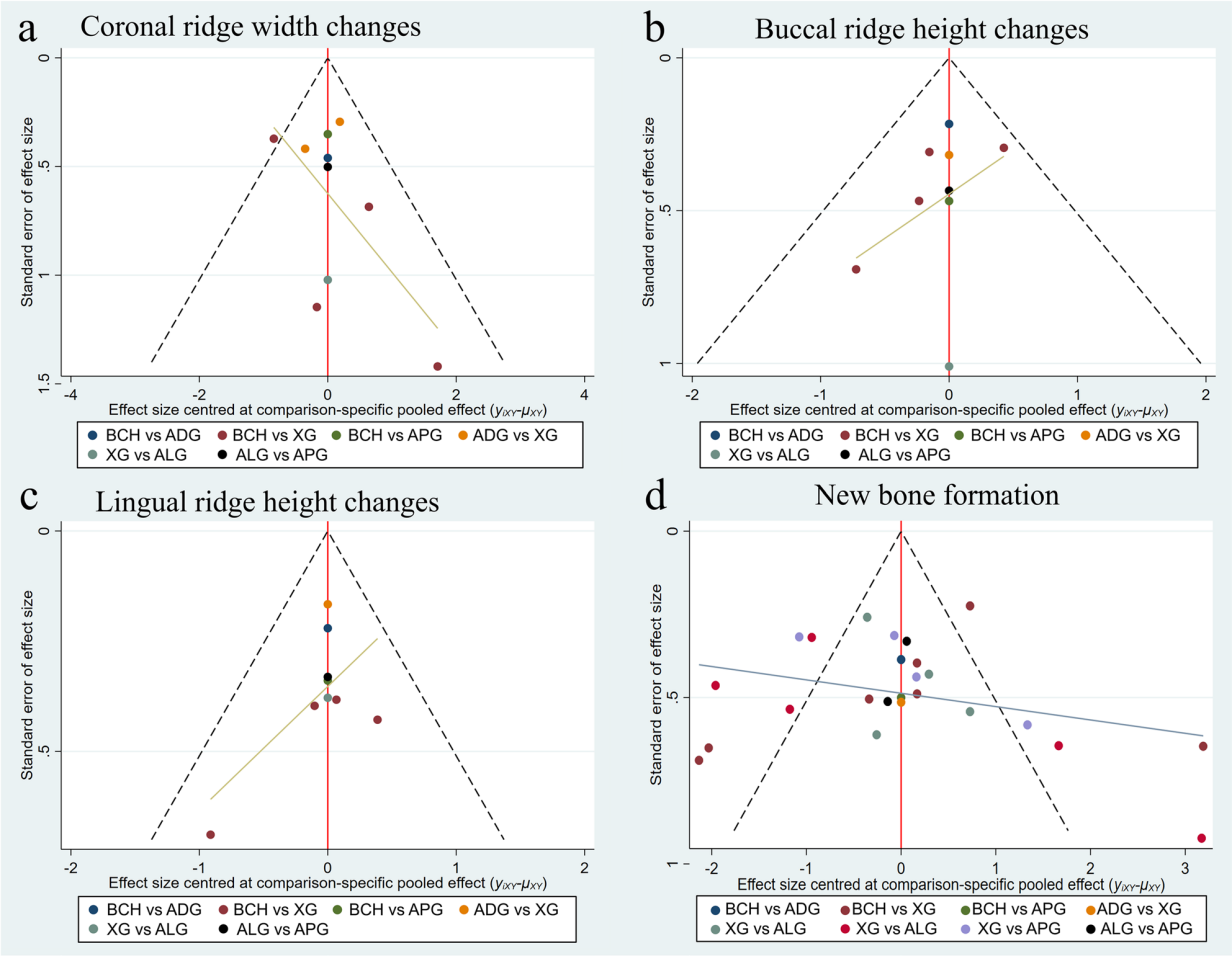
Funnel plots for coronal ridge width changes (a), buccal ridge height changes (**b**), lingual ridge height changes (**c**), and new bone formation (**d**). In the absence of publication bias and heterogeneity, the studies expected to lie within the pseudo 95% CIs and hence inverted funnel plot shape. A: blood clot healing; B: autologous dentin graft; C: xenogeneic graft; D: allogeneic graft; E: alloplastic graft

Funnel plots are used to exam the publication bias. As can be seen in Fig. 6, funnel plots for all four outcomes show good symmetry, indicating there are no small-study effects. The Egger’s test indicated there have no publication bias for all studies (Supplementary Table S7).

## Discussion

This study evaluated the comparative effectiveness of ADG versus XG, ALG, APG materials, and BCH for alveolar ridge preservation after tooth extraction. By integrating direct and indirect evidence from 25 randomized controlled trials, this network meta-analysis addresses an ADG-focused clinical question: how this not yet widely adopted autologous graft performs relative to established xenogeneic, allogeneic, and alloplastic substitutes—and to BCH—in maintaining ridge dimensions and supporting early histological healing over 3–6 months. To our knowledge, few NMAs have prespecified ADG as the index intervention while providing a broad comparison across major graft categories; Accordingly, we synthesize outcomes for coronal ridge width, buccal and lingual vertical ridge height, and histological new-bone formation.

### Summary of main findings

Across dimensional outcomes, both ADG and XG outperformed BCH for coronal ridge width and buccal ridge height, these results align with earlier studies conducted by Taschieri et al. [57] and Mahardawi et al. [58] For coronal ridge width, ADG and XG preserved, on average, 1.67 mm (95% CI: 0.81, 2.54) and 0.93 mm (95% CI: 0.20, 1.65) more width than BCH, respectively, and ADG preserved approximately 0.75 mm more than XG (95% CI:−1.45, 0.05). For buccal ridge height, ADG and XG each mitigated vertical loss relative to BCH by 0.64 mm (95% CI: 0.21, 1.08) and 0.68 mm (95% CI: 0.31, 1.04), which is consistent with the study by Zhang et al. [59] In contrast, for lingual ridge height, only XG versus BCH reached statistical significance, preserving an additional 0.69 mm (95% CI: 0.26, 1.13). Comparative rankings suggested that ADG performed best for coronal ridge width, whereas XG tended to perform best for buccal and lingual ridge height.

Histologically, ADG performed similarly to other grafts during 3–6-month follow-up period. Interpreted alongside the dimensional outcomes, these data suggest that ADG’s principal advantage is expressed in structural maintenance of post-extraction dimensions, whereas its short- to mid-term histological bone replacement appears comparable to established alternatives. Given that baseline remaining bone volume (e.g., wall count and buccal plate thickness) may modify ARP responses [60], we interpret these comparative effects in the context of typical post-extraction socket variability.

### Interpretation and mechanistic hypotheses

Anatomic context plausibly modifies ARP effects. Clinically, remaining bone volume can be operationalized by the number of intact socket walls and buccal plate thickness [61]. For clarity, we conceptualize an intact extraction socket as an intact, contained socket; partial loss of one or more walls represents partially contained or non-contained defects [62]. As emphasized by Misch and Resnik [63], the quality of the residual bony envelope governs space maintenance, blood supply, and the balance between resorption and regeneration. Within this framework, space-maintaining xenografts could exert larger relative effects in non-contained or thin-walled sites, whereas between-material differences may attenuate in well-contained five-wall sockets [64]. Even within five-wall sockets, thinner buccal plates are more prone to resorption and may benefit from strategies that emphasize scaffold stability and contour preservation (e.g., space-maintaining particulates combined with barrier membranes) [65, 66], whereas thicker plates may require less scaffold-dependent support [7]. In defects with partial wall loss, adjunctive measures such as rigid membranes, tenting, or staged augmentation may be more consequential than the specific graft substitute itself [67, 68].

The findings of this study indicate that ADG outperforms other bone substitute materials in maintaining bone volume, a superiority that we hypothesize is attributable to the following factors. Traditional bone substitute materials, subjected to high-temperature calcination or chemical processing, inevitably compromise the original bone tissue microstructure. In contrast, ADG maintains a collagen–hydroxyapatite structure and may hold bioactive non-collagen proteins (BMPs, TGF-β) that could help in bone growth, early cell joining, and tissue change [54, 69]. Its self-source might also lower body reaction. This unique biological activity, which stems from its autologous origin, virtually eliminates immunogenicity concerns while preserving complete biological functionality. Making within the xenograft group can change collagen saving and thus fixing speed. In an in vitro study, Lo Giudice [70] examined the cleanliness levels and collagen retention in equine bone blocks after steam autoclave cleaning. Although this study does not involve patient results, it suggests that collagen-retaining xenografts may behave differently than deproteinized mineral preparations during early healing.

Our findings indicate that, compared with BCH, XG more effectively preserves buccal and lingual alveolar ridge heights after tooth extraction. The height advantage of XG is largely attributable to its human bone–like trabecular architecture, which provides stable scaffolding and strong osteoconductivity for early integration, thereby resisting vertical collapse of the socket walls [71]. In addition, the typically higher material density of XG confers greater resistance to compressive deformation under occlusal and soft-tissue pressures, helping sustain vertical dimension during the critical healing phase [72]. The ability to fine-tune particle size and density further improves packing stability and vertical support in blood-rich extraction sites. Collectively, these features explain why XG excels in height preservation.

ARP is a complex biological process that extends beyond mere volume maintenance, it also emphasizes the importance of new bone formation [73, 74]. The study analyzes bone healing histologically to quantify new bone ratios, reflecting the resorption rate and osteogenic activity of bone graft substitutes. An ideal bone grafts should have a predictable resorption rate that coincides with the regeneration rate of bone tissue [75].

The findings indicated that ADG exhibited no significant differences in the process of developing new bones when contrasted with other graft materials, demonstrating bone regeneration potential similar to other grafts. While ADG has immunological and biomechanical characteristics presenting complex interactions, they do not significantly undermine its bone regeneration capacity. According to SUCRA, new bone formation efficacy of XG appears suboptimal. Slowly resorbing bone substitutes like xenogeneic particulates, may persist as residual particles during early healing, thereby reducing the histomorphometrically measured percentage of new bone even when space maintenance is adequate [76, 77]. This not only provides theoretical support for clinical decision-making but also demonstrates that ADG itself possesses substantial osteogenic potential.

### Strengths and limitations

This NMA synthesized a relatively large randomized evidence base and integrated direct and indirect comparisons across dimensional and histological outcomes, placing ADG at the center of the comparative network. Nonetheless, important limitations remain. First, clinical and methodological heterogeneity, particularly gaps in baseline data and sparse comparisons for certain contrasts (number of residual walls and buccal plate thickness), prevented meaningful subgroup analyses or meta-regression and likely introduced heterogeneity in outcomes. Second, histomorphometric processing and quantification varied across trials; although we reported absolute percentage-point differences to improve interpretability, measurement heterogeneity persists. Third, network geometry meant that several contrasts (notably involving ALG and APG) relied on indirect evidence; direct comparisons were limited. We mitigated these issues through random-effects modeling and outcome harmonization. And network rankings are best viewed as comparative summaries rather than definitive statements of superiority.

### Clinical implications and future research

Within 3–6 months, ADG and XG each mitigate early dimensional loss versus BCH; ADG appears favorable for coronal ridge width and XG trends favor buccal and lingual ridge height, with small absolute differences and moderate-to-low certainty. Given inconsistent reporting of defect anatomy, we avoid defect-specific recommendations. Material choice should be individualized (handling, availability, preferences, cost, timing, experience), with anatomy viewed as a plausible—but untested—effect modifier in this NMA.

More high-quality studies should be included in the future, prespecified morphology stratification (residual wall count, buccal plate thickness) using standardized definitions, harmonized histology, longer follow-up, systematic adverse-event reporting, and implant-level/patient-centered outcomes, ideally supported by imaging-based volumetry.

## Conclusions

In ARP, ADG reduced early post-extraction dimensional loss versus BCH and showed a relative advantage in preserving coronal ridge width compared with particulate xenogeneic, allogeneic, and alloplastic grafts. These findings support ADG as a clinically credible option, particularly when buccal or coronal width and an autologous, non-xenogeneic material are prioritized. Despite superior volume maintenance and biological compatibility, the complexity of ADG’s biological interactions necessitates further standardized, comprehensive investigations to fully elucidate their transformative potential in bone tissue reconstruction.

### Data availability

The remaining data utilized and examined in this investigation can be found in the Supplementary Information files. Additional information can be obtained by contacting the corresponding author.

### Declaration of interest statement

The authors declare that they have no conflict of interest.

## Supplementary Information

Supplementary Material 1.

## Data Availability

All data generated or analyzed during this study are included in this published article and its supplementary information files.

## Abbreviations

ADG: Autologous Particulate Dentin Graft
ALG: Allogeneic Graft
APG: Alloplastic Graft
ARP: Alveolar Ridge Preservation
BCH: Blood Clot Healing
CBCT: Cone Beam Computed Tomography
CI: Confidence Intervals
CINeMA: Confidence in Network Meta-Analysis
GRADE: Grading of Recommendations Assessment, Development, and Evaluation
MD: Mean Differences
NMA: Network Meta-Analysis
PICOS: Population, Intervention, Comparison, Outcome, and Study design
PRISMA: Preferred Reporting Items for Systematic Reviews and Meta-Analyses
RCT: Randomized Controlled Trial
SMD: Standardized Mean Difference
SUCRA: Surface Under the Cumulative Ranking Curve
XG: Particulate Xenogeneic Graft
