# Supplementary material for: Comparative effectiveness of autologous particulate dentin graft for alveolar ridge preservation: a network meta-analysis of randomized controlled trials
Source: BMC Oral Health. 2025 Dec 3;25:1914. doi: 10.1186/s12903-025-07371-7 (PMC12715967; doi:10.1186/s12903-025-07371-7)
Supplement: Supplementary file 1 — Supplementary Material 1. [file 12903_2025_7371_MOESM1_ESM.docx]

**Supplementary Material**

**Comparative Effectiveness of Autologous Particulate Dentin Graft for Alveolar Ridge Preservation: A Network Meta-Analysis of Randomized Controlled Trials**

**Supplementary Table S1** Search Strategy Used for Literature Search

**Supplementary Table S2** Reasons of excluded studies

**Supplementary Table S3** Data of outcomes

**Supplementary Table S4** GRADE Evidence Profile for each comparison

**Supplemental Table S5.** τ^2^and I² statistic for between-study variability.

**Supplementary Table S6** Test of global inconsistency of outcomes

**Supplementary Table S7** Test of publication bias

**Supplementary Table S1** Search Strategy Used for Literature Search.

| PubMed |  |
| --- | --- |
| Search Number | Search Details |
| 4 | (#1 OR #2) AND #3 |
| 3 | "alveolar ridge preservation"[All Fields] OR "socket preservation"[All Fields] OR "bone augmentation"[All Fields] OR "bone regeneration"[All Fields] |
| 2 | ("autologous dental"[Title/Abstract] OR "autogenous dentin"[Title/Abstract] OR "autologous dentin"[Title/Abstract] OR "demineralized dentin matrix"[Title/Abstract]) OR (demineralized dentin matrix[Mesh Terms]) |
| 1 | "xenograft"[Title/Abstract] OR "bone substitutes"[Title/Abstract] OR "Bio-Oss"[Title/Abstract] OR "allograft"[Title/Abstract] OR "alloplast" [Title/Abstract] OR Xenografts[Mesh Terms] OR Allograft [Mesh Terms] |

| EMBASE |  |
| --- | --- |
| No. | Query Results |
| #5. | #3 AND #4 |
| #4. | #1 OR #2 |
| #3. | 'alveolar ridge preservation'/exp OR 'alveolar ridge preservation' OR 'socket preservation' OR 'bone augmentation'/exp OR 'bone augmentation' OR 'bone regeneration'/exp OR 'bone regeneration' |
| #2. | 'autologous dental' OR 'autogenous dentin' OR 'autologous dentin' OR 'autologous particulate dentin' OR 'demineralized dentin matrix'/exp OR 'demineralized dentin matrix' |
| #1. | 'xenograft'/exp OR 'xenograft' OR 'xenogenetic graft' OR 'bone substitutes'/exp OR 'bone substitutes' OR 'bio-oss' OR 'allograft' OR 'alloplast' |

| Cochrane Library |  |
| --- | --- |
| ID | Search |
| #1 | [Heterografts] in all MeSH products |
| #2 | [Bone Substitutes] explode all trees |
| #3 | [Allografts] in all MeSH products |
| #4 | ('xenograft' OR 'xenogenetic graft' OR 'bone substitutes' OR 'Bio-Oss' OR 'allograft' OR 'alloplast'): ti,ab,kw |
| #5 | (“autologous dental” OR “autogenous dentin” OR “autologous dentin” OR “autologous particulate dentin” OR “demineralized dentin matrix”): ti,ab,kw |
| #6 | (“alveolar ridge preservation” OR “socket preservation” OR “bone augmentation” OR “bone regeneration”): ti,ab,kw |
| #7 | #1 OR #2 OR #3 OR #4 OR #5 |
| #8 | #6 AND #7 |

| Web of Science |  |
| --- | --- |
| # | Search |
| 1 | (TS=(“xenograft” OR “xenogenetic graft” OR “bone substitutes” OR “Bio-Oss” OR “allograft” OR “alloplast”)) OR (TS=(“autologous dental” OR “autogenous dentin” OR “autologous dentin” OR “autologous particulate dentin” OR “demineralized dentin matrix”)) |
| 2 | TS=(“alveolar ridge preservation” OR “socket preservation” OR “bone augmentation” OR “bone regeneration”) |
| 3 | #1 AND #2 |

| CNKI |  |
| --- | --- |
| # | Search |
| 1 | Title/Abstract/Keywords= xenograft + bone substitute + Bio-Oss + allograft + allogeneic graft |
| 2 | Title/Abstract/Keywords= autogenous dentin + demineralized dentin matrix |
| 3 | Title/Abstract/Keywords= bone augmentation + bone regeneration + alveolar ridge preservation + socket preservation |
| 4 | #1 OR #2 AND #3 |

| Wanfang |  |
| --- | --- |
| # | Search |
| 1 | Title/Abstract/Keywords= xenograft + bone substitute + Bio-Oss + allograft + allogeneic graft |
| 2 | Title/Abstract/Keywords= autogenous dentin + demineralized dentin matrix |
| 3 | Title/Abstract/Keywords= bone augmentation + bone regeneration + alveolar ridge preservation + socket preservation |
| 4 | #1 OR #2 AND #3 |
| VIP |  |
| # | Search |
| 1 | Title/Abstract/Keywords= xenograft + bone substitute + Bio-Oss + allograft + allogeneic graft+ autogenous dentin + demineralized dentin matrix |
| 2 | Title/Abstract/Keywords= bone augmentation + bone regeneration + alveolar ridge preservation + socket preservation |
| 3 | #1 AND #2 |

**Supplementary Table S2** Reasons of excluded studies

| Author | Title | Reasons for excluded |
| --- | --- | --- |
| G. Avila-Ortiz et al. (2020) | Efficacy of Alveolar Ridge Preservation: A Randomized Controlled Trial | Using non-absorbable membranes or free gingival graft as barrier membranes. |
| Parashis et al. (2016) | Prospective Clinical and Radiographic Study of Alveolar Ridge Preservation Combining FDBA With Two Xenogeneic Collagen Matrices |  |
| El-Sioufi et al. (2022) | Clinical evaluation of different alveolar ridge preservation techniques after tooth extraction: A randomized clinical trial |  |
| Kusuvan et al. (2024) | Influence of freeze-dried bone allograft on free gingival graft survival and alveolar ridge maintenance in socket seal procedures: a randomized controlled clinical trial |  |
| Santana et al. (2018) | Synthetic polymeric barrier membrane associated with blood coagulum, human allograft, or bovine bone substitute for ridge preservation: a randomized, controlled, clinical and histological trial |  |
| Isola et al. (2022) | Use of Autogenous Tooth-Derived Mineralized Dentin Matrix in the Alveolar Ridge Preservation Technique: Clinical and Histologic Evaluation |  |
| Vail et al. (2018) | Preservation of Dental Sockets Filled with Composite Bovine Bone. A Single-Blind Randomized Clinical Trial | The bone graft substitutes used in the study do not match those in our research. |
| Hu et al. (2021) | Combigraft versus Bio- Oss/Bio- Gide in alveolar ridge preservation: A prospective randomized controlled trial |  |
| Elfana et al. (2021) | Alveolar ridge preservation using autogenous whole- tooth versus demineralized dentin grafts: A randomized controlled clinical trial |  |
| Bureekanchan et al. (2024) | Competence of allogenic demineralized tooth matrix in socket seal surgery for alveolar ridge preservation: a randomized control clinical trial |  |
| Dubey et al. (2024) | Assessment of Autogenous Tooth Dentin Graft with Allogenic Bone Graft in the Preservation of Alveolar Ridge: A Clinicoradiographic Study |  |
| Wu et al. (2019) | Short-term Clinical Outcomes of Autogenous Tooth Bone Graft Material Application in Implant Surgery |  |
| Keil et al. (2021) | Histological evaluation of extraction sites grafted with Bio-Oss Collagen: Randomized controlled trial | The follow-up period is less than 3 months or longer than 6 months. |
| Jonker et al. (2020) | Soft tissue contour and radiographic evaluation of ridge preservation in early implant placement: A randomized controlled clinical trial |  |
| Mendez et al. (2017) | Comparison of allografts and xenografts used for alveolar ridge preservation. A clinical and histomorphometric RCT in humans | Bone dimensions were measured with a periodontal probe during operations. |
| Sbordone et al. (2017) | Evaluation of volumetric dimensional changes in posterior extraction sites with and without ARP using a novel imaging device |  |
| Stefan et al. (2017) | Dimensional evaluation of different ridge preservation techniques: a randomized clinical study |  |
| Guarnieri et al. (2017) | Extraction Socket Preservation Using Porcine-Derived Collagen Membrane Alone or Associated with Porcine-Derived Bone. Clinical Results of Randomized Controlled Study |  |
| Sacristán et al. (2024) | Use of autologous tooth-derived material as a graft in the post-extraction socket. Split-mouth study with radiological and histological analysis | No mentioning newly formed bone or changes in vertical and horizontal bone dimensions. |
| Khouly et al. (2021) | Effect of alveolar ridge preservation on clinical attachment level at adjacent teeth: A randomized clinical trial |  |
| Fischer et al. (2018) | Dimensional evaluation of different ridge preservation techniques with a bone xenograft: a randomized controlled clinical trial |  |
| Beldhi et al. (2024) | Evaluation and comparison of autologous particulate dentin with demineralized freeze dried bone allograft in ridge preservation procedures – A prospective clinical study |  |
| MacBeth et al. (2024) | Healing patterns of alveolar bone following ridge preservation procedures | Presented patients or data repeated in other articles included. |
| Ikhlas Abd Dkheel et al. (2023) | The Effectiveness of Autogenous Dentin Graft as a Biomaterial in Optimizing the Esthetic Outcomes of Immediate Dental Implants: A Prospective Clinical Study | Not alveolar ridge preservation interventions |
| Wu et al. (2025) | Clinical efficacy of demineralized dentin matrix particles in immediate implantation for bone defects in posterior region: a 1 to 5- year follow-up study |  |
| Meng et al. (2021) | Horizontal bone augmentation in the posterior mandible by means of GBR with resorbable collagen membrane and particulate xenograft |  |

**Supplementary Table S3** Main results of included studies

| **Study** | **Group** | **Coronal horizontal ridge width** | | | **Buccal Crest Height** | | | **Lingual Crest Height** | | | **New Bone Formation** | | |
| --- | --- | --- | --- | --- | --- | --- | --- | --- | --- | --- | --- | --- | --- |
|  |  | N | MD | SD | N | MD | SD | N | MD | SD | N | MD | SD |
| Abellán et al, 2022[1] | XG | 11 | -3.2 | 2.39 | 11 | -1.76 | 1.72 | 11 | -1.13 | 1.13 | 7 | 46.44 | 16.49 |
|  | ALG | 10 | -2.73 | 2.29 | 10 | -2.21 | 2.74 | 10 | -1.33 | 0.52 | 7 | 48.54 | 18.78 |
| Barone et al, 2016[2] | BCH | NR | NR | NR | NR | NR | NR | NR | NR | NR | 30 | 44 | 14.7 |
|  | XG | NR | NR | NR | NR | NR | NR | NR | NR | NR | 60 | 39.1 | 19.8 |
| Cadenas-Vacas et al,2021[3] | XG | NR | NR | NR | NR | NR | NR | NR | NR | NR | 12 | 11 | 7 |
|  | APG | NR | NR | NR | NR | NR | NR | NR | NR | NR | 12 | 23 | 15 |
| Candrli´ et al, 2022[4] | XG | NR | NR | NR | NR | NR | NR | NR | NR | NR | 20 | 30.47 | 16.39 |
|  | APG | NR | NR | NR | NR | NR | NR | NR | NR | NR | 20 | 26.47 | 14.72 |
| Carlos et al, 2017[5] | XG | NR | NR | NR | NR | NR | NR | NR | NR | NR | 10 | 35.3 | 16.8 |
|  | ALG | NR | NR | NR | NR | NR | NR | NR | NR | NR | 10 | 25.5 | 10.1 |
| Casarez-Quintana et al,2021[6] | XG | NR | NR | NR | NR | NR | NR | NR | NR | NR | 23 | 26.8 | 15.6 |
|  | APG | NR | NR | NR | NR | NR | NR | NR | NR | NR | 21 | 39.31 | 17.1 |
| Gabay et al, 2022[7] | BCH | NR | NR | NR | NR | NR | NR | NR | NR | NR | 14 | 51.14 | 23.04 |
|  | XG | NR | NR | NR | NR | NR | NR | NR | NR | NR | 14 | 33.79 | 17.37 |
| Guarnieri et al,2017[8] | BCH | NR | NR | NR | NR | NR | NR | NR | NR | NR | 9 | 48.85 | 2.37 |
|  | XG | NR | NR | NR | NR | NR | NR | NR | NR | NR | 8 | 57.43 | 4.8 |
| Hussain et al,2023[9] | BCH | 15 | -3.54 | 1.26 | 15 | -0.84 | 0.83 | 15 | -0.71 | 0.82 | NR | NR | NR |
|  | ADG | 14 | -1.47 | 1.22 | 14 | -0.31 | 0.11 | 14 | -0.27 | 0.23 | NR | NR | NR |
| Jung et al, 2018A[10] | XG | 8 | -1.68 | 1.11 | 8 | -1.14 | 0.81 | 8 | -0.65 | 0.37 | 6 | 22 | 11.01 |
|  | ADG | 8 | -0.78 | 0.41 | 8 | -0.97 | 0.39 | 8 | -0.76 | 0.29 | 6 | 32.88 | 14.48 |
| Junget al ,2018B[11] | BCH | 18 | -3 | 2.63 | 18 | -1.39 | 1.14 | 18 | -1.04 | 1.59 | NR | NR | NR |
|  | XG | 18 | -1.39 | 1.24 | 18 | -0.22 | 0.51 | 18 | -0.24 | 0.55 | NR | NR | NR |
| Kim et al,2024[12] | BCH | 17 | -0.84 | 3.63 | 17 | -0.64 | 1.73 | 17 | -0.43 | 1.61 | NR | NR | NR |
|  | XG | 17 | -0.04 | 3.03 | 17 | -0.62 | 2.27 | 17 | -0.44 | 2.34 | NR | NR | NR |
| Lim et al,2019[13] | BCH | 8 | -4.44 | 3.71 | 8 | -1.33 | 1.11 | 8 | -1.2 | 0.96 | 6 | 25.16 | 18.45 |
|  | XG | 21 | -1.76 | 2.49 | 21 | -0.82 | 1.17 | 21 | -0.23 | 0.81 | 18 | 13.81 | 11.32 |
| MacBeth et al,2022[14] | BCH | 14 | -2.3 | 1.11 | 14 | -0.52 | 0.8 | 14 | -0.43 | 0.83 | NR | NR | NR |
|  | XG | 14 | -2.17 | 0.84 | 14 | 0.07 | 0.83 | 14 | 0.86 | 1.37 | NR | NR | NR |
| Machtei et al,2019[15] | BCH | NR | NR | NR | NR | NR | NR | NR | NR | NR | 10 | 81.72 | 4.3 |
|  | XG | NR | NR | NR | NR | NR | NR | NR | NR | NR | 11 | 22.5 | 24.72 |
| Nunes et al, 2018[16] | BCH | 15 | -1.3 | 1.22 | 15 | -1.66 | 1.37 | 15 | -0.26 | 0.79 | NR | NR | NR |
|  | APG | 15 | -0.55 | 0.6 | 15 | -1.57 | 1.19 | 15 | -0.44 | 1.05 | NR | NR | NR |
| Ogui´c et al ,2023[17] | ADG | 20 | -0.88 | 0.76 | NR | NR | NR | NR | NR | NR | 12 | 72.55 | 12.14 |
|  | XG | 17 | -1.24 | 0.99 | NR | NR | NR | NR | NR | NR | 10 | 69.61 | 13.53 |
| Pang et al, 2017[18] | ADG | NR | NR | NR | NR | NR | NR | NR | NR | NR | 8 | 31.24 | 13.87 |
|  | XG | NR | NR | NR | NR | NR | NR | NR | NR | NR | 6 | 35 | 19.33 |
| Sadeghi et al, 2015[19] | XG | NR | NR | NR | NR | NR | NR | NR | NR | NR | 10 | 18.76 | 3.54 |
|  | ALG | NR | NR | NR | NR | NR | NR | NR | NR | NR | 10 | 34.49 | 3.19 |
| Saito et al, 2021[20] | ALG | 17 | -1.28 | 1.73 | 17 | -0.57 | 1.44 | 17 | -0.85 | 1 | 17 | 38.2 | 12..5 |
|  | APG | 23 | -1.26 | 1.32 | 23 | -0.53 | 1.24 | 23 | -0.24 | 1.08 | 22 | 27 | 22.1 |
| Santos et al, 2021[21] | ADG | NR | NR | NR | NR | NR | NR | NR | NR | NR | 34 | 47.3 | 14.8 |
|  | XG | NR | NR | NR | NR | NR | NR | NR | NR | NR | 32 | 34.9 | 13.2 |
| Scheyer et al, 2016[22] | XG | NR | NR | NR | NR | NR | NR | NR | NR | NR | 19 | 29.81 | 9.03 |
|  | ALG | NR | NR | NR | NR | NR | NR | NR | NR | NR | 21 | 33.36 | 11.09 |
| Stumbras et al,2020[23] | BCH | NR | NR | NR | NR | NR | NR | NR | NR | NR | 10 | 46.5 | 15.2 |
|  | XG | NR | NR | NR | NR | NR | NR | NR | NR | NR | 10 | 20.3 | 21.9 |
| Yu¨ceer-C¸etiner et al, 2021[24] | BCH | NR | NR | NR | NR | NR | NR | NR | NR | NR | 12 | 18.68 | 1.18 |
|  | ADG | NR | NR | NR | NR | NR | NR | NR | NR | NR | 16 | 19.32 | 1.91 |
| Zampara et al,2022[25] | BCH | NR | NR | NR | NR | NR | NR | NR | NR | NR | 8 | 57 | 12.8 |
|  | XG | NR | NR | NR | NR | NR | NR | NR | NR | NR | 8 | 17.2 | 12.8 |
|  | ALG | NR | NR | NR | NR | NR | NR | NR | NR | NR | 8 | 56 | 12.8 |
|  | APG | NR | NR | NR | NR | NR | NR | NR | NR | NR | 8 | 45.6 | 12.8 |
| N: number of sample size; MD: mean difference; SD: standard deviation; ADG: autologous particulate dentin graft; ALG: allogeneic graft; APG: alloplastic graft; BCH: blood clot healing; NR: not reported. | | | | | | | | | | | | | |

**Supplementary Table S4** GRADE Evidence Profile for each comparison. ADG: autologous particulate dentin graft; ALG: allogeneic graft; APG: alloplastic graft; BCH: blood clot healing.

**Question:** Should ADG vs BCH be used for Alveolar Ridge Preservation?

| **Quality assessment** | | | | | | | **No of patients** | | **Effect** | | **Quality** | **Importance** |  |
| --- | --- | --- | --- | --- | --- | --- | --- | --- | --- | --- | --- | --- | --- |
|  |  |  |  |  |  |  |  |  |  |  |  |  |  |
| **No of studies** | **Design** | **Risk of bias** | **Inconsistency** | **Indirectness** | **Imprecision** | **Other considerations** | **ADG** | **BCH** | **Relative (95% CI)** | **Absolute** |  |  |  |
| **The vertical buccal crest height (follow-up mean 4 months; Better indicated by lower values)** | | | | | | | | | | | | |  |
| 1 | randomised trials | no serious risk of bias | no serious inconsistency | no serious indirectness | serious^1^ | none | 14 | 15 | - | SMD 0.85 lower (0.09 to 1.62 higher) | ⊕⊕⊕O MODERATE | CRITICAL |  |
| **The vertical lingual crest height (follow-up mean 4 months; Better indicated by lower values)** | | | | | | | | | | | | |  |
| 1 | randomised trials | no serious risk of bias | no serious inconsistency | no serious indirectness | serious^1^ | none | 14 | 15 | - | MD 0.44 lower (0.01 to 0.87 higher) | ⊕⊕⊕O MODERATE | CRITICAL |  |
| **Horizontal alveolar ridge bone width (follow-up mean 4 months; Better indicated by lower values)** | | | | | | | | | | | | |  |
| 1 | randomised trials | no serious risk of bias | no serious inconsistency | no serious indirectness | serious^1^ | none | 14 | 15 | - | MD 2.07 higher (1.17 to 2.97 higher) | ⊕⊕⊕O MODERATE | CRITICAL |  |
| **New bone formation (follow-up mean 3 months; Better indicated by lower values)** | | | | | | | | | | | | |  |
| 1 | randomised trials | no serious risk of bias | no serious inconsistency | no serious indirectness | serious^1^ | none | 16 | 12 | - | MD 0.64 higher (0.51 lower to 1.79 higher) | ⊕⊕⊕O MODERATE | CRITICAL |  |

^1^ Downgraded one level due to imprecision: Due to the inclusion of only one literature and the small sample size, the precision of the effect estimate is insufficient.

**Question:** Should ADG vs XG be used for Alveolar Ridge Preservation?

| **Quality assessment** | | | | | | | **No of patients** | | **Effect** | | **Quality** | **Importance** |  |
| --- | --- | --- | --- | --- | --- | --- | --- | --- | --- | --- | --- | --- | --- |
|  |  |  |  |  |  |  |  |  |  |  |  |  |  |
| **No of studies** | **Design** | **Risk of bias** | **Inconsistency** | **Indirectness** | **Imprecision** | **Other considerations** | **ADG** | **XG** | **Relative (95% CI)** | **Absolute** |  |  |  |
| **Horizontal alveolar ridge bone width (follow-up mean 4 months; Better indicated by lower values)** | | | | | | | | | | | | |  |
| 2 | randomised trials | serious^1^ | no serious inconsistency | no serious indirectness | serious^2^ | none | 28 | 25 | - | MD 0.55 higher (0.04 to 1.05 higher) | ⊕⊕OO LOW | CRITICAL |  |
| **New bone formation (follow-up 4-6 months; Better indicated by lower values)** | | | | | | | | | | | | |  |
| 4 | randomised trials | serious^1^ | no serious inconsistency | no serious indirectness | no serious imprecision | none | 60 | 54 | - | MD 7.84 higher (1.25 to 14.43 higher) | ⊕⊕⊕O MODERATE | CRITICAL |  |

^1^ Downgraded one level due to risk of bias: one study at some concerns; and original review highlighted risk of bias as key issue.
^2^ Downgraded one level due to imprecision: Due to the inclusion of only one literature and the small sample size, the precision of the effect estimate is insufficient.

**Question:** Should XG vs BCH be used for Alveolar Ridge Preservation?

| **Quality assessment** | | | | | | | **No of patients** | | **Effect** | | **Quality** | **Importance** |  |
| --- | --- | --- | --- | --- | --- | --- | --- | --- | --- | --- | --- | --- | --- |
|  |  |  |  |  |  |  |  |  |  |  |  |  |  |
| **No of studies** | **Design** | **Risk of bias** | **Inconsistency** | **Indirectness** | **Imprecision** | **Other considerations** | **XG** | **BCH** | **Relative (95% CI)** | **Absolute** |  |  |  |
| **Vertical buccal crest height (follow-up 2-8 months; Better indicated by lower values)** | | | | | | | | | | | | |  |
| 4 | randomised trials | serious^1^ | no serious inconsistency | no serious indirectness | serious^2^ | none | 70 | 57 | - | MD 0.29 higher (0.66 lower to 1.25 higher) | ⊕⊕OO LOW | IMPORTANT |  |
| **The vertical lingual crest height (follow-up 2-8 months; Better indicated by lower values)** | | | | | | | | | | | | |  |
| 4 | randomised trials | serious^1^ | no serious inconsistency | no serious indirectness | serious^2^ | none | 70 | 57 | - | MD 0.13 higher (0.97 lower to 1.24 higher) | ⊕⊕OO LOW | IMPORTANT |  |
| **Horizontal alveolar ridge bone width (follow-up 2-8 months; Better indicated by lower values)** | | | | | | | | | | | | |  |
| 4 | randomised trials | serious^1^ | no serious inconsistency | no serious indirectness | serious^2^ | none | 70 | 57 | - | MD 0.97 higher (0.11 lower to 2.05 higher) | ⊕⊕OO LOW | IMPORTANT |  |
| **New bone formation (follow-up 4-6 months; Better indicated by lower values)** | | | | | | | | | | | | |  |
| 8 | randomised trials | serious^1^ | no serious inconsistency | no serious indirectness | no serious imprecision | none | 174 | 132 | - | MD 15.61 lower (29.5 to 1.72 lower) | ⊕⊕⊕O MODERATE | IMPORTANT |  |

^1^ Downgraded one level due to risk of bias: 3/5 studies at some concerns; and original review highlighted risk of bias as key issue.
^2^ Downgraded one level due to imprecision: the included studies had a wide range of follow-up durations, which may affect the consistency of the effect estimates, and the precision of the results remains uncertain.

**Question:** Should XG vs ALG be used for Alveolar Ridge Preservation?

| **Quality assessment** | | | | | | | **No of patients** | | **Effect** | | **Quality** | **Importance** |  |
| --- | --- | --- | --- | --- | --- | --- | --- | --- | --- | --- | --- | --- | --- |
|  |  |  |  |  |  |  |  |  |  |  |  |  |  |
| **No of studies** | **Design** | **Risk of bias** | **Inconsistency** | **Indirectness** | **Imprecision** | **Other considerations** | **XG** | **ALG** | **Relative (95% CI)** | **Absolute** |  |  |  |
| **The vertical buccal crest height (follow-up mean 5 months; Better indicated by lower values)** | | | | | | | | | | | | |  |
| 1 | randomised trials | serious^1^ | no serious inconsistency | no serious indirectness | serious^2^ | none | 11 | 10 | - | MD 0.47 lower (2.47 lower to 1.53 higher) | ⊕⊕OO LOW | IMPORTANT |  |
| **The vertical lingual crest height (follow-up mean 5 months; Better indicated by lower values)** | | | | | | | | | | | | |  |
| 1 | randomised trials | serious^1^ | no serious inconsistency | no serious indirectness | serious^2^ | none | 11 | 10 | - | MD 0.2 higher (0.54 lower to 0.94 higher) | ⊕⊕OO LOW | IMPORTANT |  |
| **Horizontal alveolar ridge bone width (follow-up mean 5 months; Better indicated by lower values)** | | | | | | | | | | | | |  |
| 1 | randomised trials | serious^1^ | no serious inconsistency | no serious indirectness | serious^2^ | none | 11 | 10 | - | MD 0.47 lower (2.47 lower to 1.53 higher) | ⊕⊕OO LOW | IMPORTANT |  |
| **New bone formation (follow-up 3-5 months; Better indicated by lower values)** | | | | | | | | | | | | |  |
| 3 | randomised trials | serious^1^ | no serious inconsistency | no serious indirectness | no serious imprecision | none | 34 | 36 | - | MD 14.95 lower (39.04 lower to 9.13 higher) | ⊕⊕⊕O MODERATE | IMPORTANT |  |

^1^ Downgraded one level due to risk of bias: one study at some concerns; and original review highlighted risk of bias as key issue.
^2^ Downgraded one level due to imprecision: Due to the inclusion of only one literature and the small sample size, the precision of the effect estimate is insufficient.

**Question:** Should APG vs BCH be used for Alveolar Ridge Preservation?

| **Quality assessment** | | | | | | | **No of patients** | | **Effect** | | **Quality** | **Importance** |  |
| --- | --- | --- | --- | --- | --- | --- | --- | --- | --- | --- | --- | --- | --- |
|  |  |  |  |  |  |  |  |  |  |  |  |  |  |
| **No of studies** | **Design** | **Risk of bias** | **Inconsistency** | **Indirectness** | **Imprecision** | **Other considerations** | **APG** | **BCH** | **Relative (95% CI)** | **Absolute** |  |  |  |
| **The vertical buccal crest height (follow-up mean 6 months; Better indicated by lower values)** | | | | | | | | | | | | |  |
| 1 | randomised trials | no serious risk of bias | no serious inconsistency | no serious indirectness | serious^1^ | none | 15 | 15 | - | MD 0.09 higher (0.83 lower to 1.01 higher) | ⊕⊕⊕O MODERATE | IMPORTANT |  |
| **The vertical lingual crest height (follow-up mean 6 months; Better indicated by lower values)** | | | | | | | | | | | | |  |
| 1 | randomised trials | no serious risk of bias | no serious inconsistency | no serious indirectness | serious^1^ | none | 15 | 15 | - | MD 0.18 lower (0.84 lower to 0.48 higher) | ⊕⊕⊕O MODERATE | IMPORTANT |  |
| **Horizontal alveolar ridge bone width (follow-up mean 6 months; Better indicated by lower values)** | | | | | | | | | | | | |  |
| 1 | randomised trials | no serious risk of bias | no serious inconsistency | no serious indirectness | serious^1^ | none | 15 | 15 | - | MD 0.75 higher (0.06 to 1.44 higher) | ⊕⊕⊕O MODERATE | IMPORTANT |  |
| **New bone formation (follow-up mean 3 months; Better indicated by lower values)** | | | | | | | | | | | | |  |
| 1 | randomised trials | no serious risk of bias | no serious inconsistency | no serious indirectness | serious^1^ | none | 8 | 8 | - | MD 11.4 lower (23.94 lower to 1.14 higher) | ⊕⊕⊕O MODERATE | IMPORTANT |  |

^1^ Downgraded one level due to imprecision: Due to the inclusion of only one literature and the small sample size, the precision of the effect estimate is insufficient.

**Question:** Should ALG vs APG be used for Alveolar Ridge Preservation?

| **Quality assessment** | | | | | | | **No of patients** | | **Effect** | | **Quality** | **Importance** |  |
| --- | --- | --- | --- | --- | --- | --- | --- | --- | --- | --- | --- | --- | --- |
|  |  |  |  |  |  |  |  |  |  |  |  |  |  |
| **No of studies** | **Design** | **Risk of bias** | **Inconsistency** | **Indirectness** | **Imprecision** | **Other considerations** | **ALG** | **APG** | **Relative (95% CI)** | **Absolute** |  |  |  |
| **The vertical buccal crest height (follow-up mean 4 months; Better indicated by lower values)** | | | | | | | | | | | | |  |
| 1 | randomised trials | serious^1^ | no serious inconsistency | no serious indirectness | serious^2^ | none | 23 | 17 | - | MD 0.04 higher (0.81 lower to 0.89 higher) | ⊕⊕OO LOW | IMPORTANT |  |
| **The vertical lingual crest height (follow-up mean 4 months; Better indicated by lower values)** | | | | | | | | | | | | |  |
| 1 | randomised trials | serious^1^ | no serious inconsistency | no serious indirectness | serious^2^ | none | 23 | 17 | - | MD 0.61 higher (0.04 lower to 1.26 higher) | ⊕⊕OO LOW |  |  |
| **Horizontal alveolar ridge bone width (follow-up mean 4 months; Better indicated by lower values)** | | | | | | | | | | | | |  |
| 1 | randomised trials | serious^1^ | no serious inconsistency | no serious indirectness | serious^2^ | none | 23 | 17 | - | MD 0.02 higher (0.96 lower to 1 higher) | ⊕⊕OO LOW |  |  |
| **New bone formation (follow-up 3-4 months; Better indicated by lower values)** | | | | | | | | | | | | |  |
| 2 | randomised trials | serious^1^ | no serious inconsistency | no serious indirectness | serious^2^ | none | 30 | 25 | - | MD 10.85 lower (19.12 to 2.59 lower) | ⊕⊕OO LOW |  |  |

^1^ Downgraded one level due to risk of bias: one study at some concerns; and original review highlighted risk of bias as key issue.
^2^ Downgraded one level due to imprecision: Due to the inclusion of only one or two literature and the small sample size, the precision of the effect estimate is insufficient.

**Question:** Should XG vs APG be used for Alveolar Ridge Preservation?

| **Quality assessment** | | | | | | | **No of patients** | | **Effect** | | **Quality** | **Importance** |  |
| --- | --- | --- | --- | --- | --- | --- | --- | --- | --- | --- | --- | --- | --- |
|  |  |  |  |  |  |  |  |  |  |  |  |  |  |
| **No of studies** | **Design** | **Risk of bias** | **Inconsistency** | **Indirectness** | **Imprecision** | **Other considerations** | **XG** | **APG** | **Relative (95% CI)** | **Absolute** |  |  |  |
| **New bone formation (follow-up mean 3 months; Better indicated by lower values)** | | | | | | | | | | | | |  |
| 4 | randomised trials | serious^1^ | no serious inconsistency | no serious indirectness | no serious imprecision | none | 63 | 61 | - | MD 11.81 lower (23.87 lower to 0.26 higher) | ⊕⊕⊕O MODERATE | IMPORTANT |  |

^1^ Downgraded one level due to risk of bias: two studies at some concerns; and original review highlighted risk of bias as key issue.

| **Supplemental Table S5.** τ^2^and I² statistic for between-study variability. | | | | |
| --- | --- | --- | --- | --- |
| Comparative Combination | Coronal ridge width change | Buccal ridge height changes | Lingual ridge height changes | New bone formaiton |
| BCH vs ADG | NA | NA | NA | NA |
| XG vs ADG | 0.02, 10% | NA | NA | 0.08, 29% |
| BCH vs XG | 0.57, 50% | 0.03, 18% | 0.00, 0% | 1.39, 87% |
| ALG vs XG | NA | NA | NA | 2.21, 89% |
| BCH vs APG | NA | NA | NA | NA |
| ALG vs APG | NA | NA | NA | 0.00, 0% |
| XG vs APG | NR | NR | NR | 0.54, 78% |
| Heterogeneity is presented as: τ^2^, I². NA, not applicable: heterogeneity not assessable with single study. NR, not reported: no data for this comparison. BCH: blood clot healing; XG: xenogeneic graft; ADG: autologous dentin graft; ALG: allogeneic graft; APG: alloplastic graft. | | | | |

**Supplementary Table S6** Test of global inconsistency of outcomes.

| **Network outcome** | **P-value for test of global inconsistency** |
| --- | --- |
| Coronal ridge width changes | 0.735 |
| Buccal ridge height changes | 0.784 |
| Lingual ridge height changes | 0.060 |
| New bone formation | 0.709 |

**Supplementary Table S7** Test of publication bias.

| **Network outcome** | **P-value for test of publication bias** |
| --- | --- |
| Coronal ridge width changes | 0.222 |
| Buccal ridge height changes | 0.229 |
| Lingual ridge height changes | 0.373 |
| New bone formation | 0.416 |

**References**

1. Abellán D, Barallat L, Vilarrasa J, Cabezas M, Pascual La Rocca A, Valles C and Nart J (2022) Ridge preservation in molar sites comparing xenograft versus mineralized freeze-dried bone allograft: A randomized clinical trial. Clin Oral Implants Res 33:511-523. doi: 10.1111/clr.13911

2. Barone A, Toti P, Quaranta A, Alfonsi F, Cucchi A, Negri B, Di Felice R, Marchionni S, Calvo-Guirado JL, Covani U and Nannmark U (2017) Clinical and Histological changes after ridge preservation with two xenografts: preliminary results from a multicentre randomized controlled clinical trial. J Clin Periodontol 44:204-214. doi: 10.1111/jcpe.12655

3. Cadenas-Vacas G, Martínez-Rodríguez N, Barona-Dorado C, Sánchez-Labrador L, Cortés-Bretón Brinkmann J, Meniz-García C and Martínez-González JM (2021) Calcium Phosphate Modified with Silicon vs. Bovine Hydroxyapatite for Alveolar Ridge Preservation: Densitometric Evaluation, Morphological Changes and Histomorphometric Study. Materials (Basel, Switzerland) 14. doi: 10.3390/ma14040940

4. Čandrlić M, Tomas M, Karl M, Malešić L, Včev A, Perić Kačarević Ž and Matijević M (2022) Comparison of Injectable Biphasic Calcium Phosphate and a Bovine Xenograft in Socket Preservation: Qualitative and Quantitative Histologic Study in Humans. Int J Mol Sci 23. doi: 10.3390/ijms23052539

5. Serrano Méndez CA, Lang NP, Caneva M, Ramírez Lemus G, Mora Solano G and Botticelli D (2017) Comparison of allografts and xenografts used for alveolar ridge preservation. A clinical and histomorphometric RCT in humans. Clin Implant Dent Relat Res 19:608-615. doi: 10.1111/cid.12490

6. Casarez-Quintana A, Mealey BL, Kotsakis G and Palaiologou A (2022) Comparing the histological assessment following ridge preservation using a composite bovine-derived xenograft versus an alloplast hydroxyapatite-sugar cross-linked collagen matrix. J Periodontol 93:1691-1700. doi: 10.1002/jper.22-0149

7. Gabay E, Katorza A, Zigdon‐Giladi H, Horwitz J, Machtei EEJCid and research r (2022) Histological and dimensional changes of the alveolar ridge following tooth extraction when using collagen matrix and collagen‐embedded xenogenic bone substitute: A randomized clinical trial. 24:382-390.

8. Guarnieri R, Testarelli L, Stefanelli L, De Angelis F, Mencio F, Pompa G and Di Carlo S (2017) Bone Healing in Extraction Sockets Covered With Collagen Membrane Alone or Associated With Porcine-Derived Bone Graft: a Comparative Histological and Histomorphometric Analysis. Journal of oral & maxillofacial research 8:e4. doi: 10.5037/jomr.2017.8404

9. Hussain AA, Al-Quisi AF and Abdulkareem AA (2023) Efficacy of Autogenous Dentin Biomaterial on Alveolar Ridge Preservation: A Randomized Controlled Clinical Trial. Biomed Res Int 2023:7932432. doi: 10.1155/2023/7932432

10. Jung G-U, Jeon T-H, Kang M-H, Um I-W, Song I-S, Ryu J-J and Jun S-HJAS (2018) Volumetric, radiographic, and histologic analyses of demineralized dentin matrix combined with recombinant human bone morphogenetic protein-2 for ridge preservation: a prospective randomized controlled trial in comparison with xenograft. 8:1288.

11. Jung RE, Sapata VM, Hämmerle CHF, Wu H, Hu XL and Lin Y (2018) Combined use of xenogeneic bone substitute material covered with a native bilayer collagen membrane for alveolar ridge preservation: A randomized controlled clinical trial. Clin Oral Implants Res 29:522-529. doi: 10.1111/clr.13149

12. Kim H, Han HS, Ghanaati S, Zadeh HH, Kim S and Cho YD (2024) Alveolar Ridge Preservation Using a Collagenated Xenograft: A Randomized Clinical Trial. Int Dent J. doi: 10.1016/j.identj.2024.07.015

13. Lim HC, Shin HS, Cho IW, Koo KT and Park JC (2019) Ridge preservation in molar extraction sites with an open-healing approach: A randomized controlled clinical trial. J Clin Periodontol 46:1144-1154. doi: 10.1111/jcpe.13184

14. MacBeth ND, Donos N and Mardas N (2022) Alveolar ridge preservation with guided bone regeneration or socket seal technique. A randomised, single-blind controlled clinical trial. Clin Oral Implants Res 33:681-699. doi: 10.1111/clr.13933

15. Machtei EE, Mayer Y, Horwitz J and Zigdon-Giladi H (2019) Prospective randomized controlled clinical trial to compare hard tissue changes following socket preservation using alloplasts, xenografts vs no grafting: Clinical and histological findings. Clin Implant Dent Relat Res 21:14-20. doi: 10.1111/cid.12707

16. Nunes FAS, Pignaton TB, Novaes AB, Jr., Taba M, Jr., Messora MR, Palioto DB, Spin-Neto R and de Souza SLS (2018) Evaluation of a bone substitute covered with a collagen membrane for ridge preservation after tooth extraction. Clinical and tomographic randomized controlled study in humans. Clin Oral Implants Res 29:424-433. doi: 10.1111/clr.13140

17. Oguić M, Čandrlić M, Tomas M, Vidaković B, Blašković M, Jerbić Radetić AT, Zoričić Cvek S, Kuiš D and Cvijanović Peloza O (2023) Osteogenic Potential of Autologous Dentin Graft Compared with Bovine Xenograft Mixed with Autologous Bone in the Esthetic Zone: Radiographic, Histologic and Immunohistochemical Evaluation. Int J Mol Sci 24. doi: 10.3390/ijms24076440

18. Pang KM, Um IW, Kim YK, Woo JM, Kim SM and Lee JH (2017) Autogenous demineralized dentin matrix from extracted tooth for the augmentation of alveolar bone defect: a prospective randomized clinical trial in comparison with anorganic bovine bone. Clin Oral Implants Res 28:809-815. doi: 10.1111/clr.12885

19. Sadeghi R, Babaei M, Miremadi SA and Abbas FM (2016) A randomized controlled evaluation of alveolar ridge preservation following tooth extraction using deproteinized bovine bone mineral and demineralized freeze-dried bone allograft. Dent Res J (Isfahan) 13:151-9. doi: 10.4103/1735-3327.178202

20. Saito H, Couso-Queiruga E, Shiau HJ, Stuhr S, Prasad H, Allareddy TV, Reynolds MA and Avila-Ortiz G (2021) Evaluation of poly lactic-co-glycolic acid-coated β-tricalcium phosphate for alveolar ridge preservation: A multicenter randomized controlled trial. J Periodontol 92:524-535. doi: 10.1002/jper.20-0360

21. Santos A, Botelho J, Machado V, Borrecho G, Proença L, Mendes JJ, Mascarenhas P and Alcoforado G (2021) Autogenous Mineralized Dentin versus Xenograft granules in Ridge Preservation for Delayed Implantation in Post-extraction Sites: A Randomized controlled clinical trial with an 18 months follow-up. Clin Oral Implants Res 32:905-915. doi: 10.1111/clr.13765

22. Scheyer ET, Heard R, Janakievski J, Mandelaris G, Nevins ML, Pickering SR, Richardson CR, Pope B, Toback G, Velásquez D and Nagursky H (2016) A randomized, controlled, multicentre clinical trial of post-extraction alveolar ridge preservation. J Clin Periodontol 43:1188-1199. doi: 10.1111/jcpe.12623

23. Stumbras A, Januzis G, Gervickas A, Kubilius R and Juodzbalys G (2020) Randomized and Controlled Clinical Trial of Bone Healing After Alveolar Ridge Preservation Using Xenografts and Allografts Versus Plasma Rich in Growth Factors. The Journal of oral implantology 46:515-525. doi: 10.1563/aaid-joi-D-19-00179

24. Yüceer-Çetiner E, Özkan N and Önger ME (2021) Effect of Autogenous Dentin Graft on New Bone Formation. The Journal of craniofacial surgery 32:1354-1360. doi: 10.1097/scs.0000000000007403

25. Zampara E, Alshammari M, De Bortoli J, Mullings O, Gkisakis IG, Benalcázar Jalkh EB, Tovar N, Coelho PG and Witek L (2022) A Histologic and Histomorphometric Evaluation of an Allograft, Xenograft, and Alloplast Graft for Alveolar Ridge Preservation in Humans: A Randomized Controlled Clinical Trial. The Journal of oral implantology 48:541-549. doi: 10.1563/aaid-joi-D-21-00012
